# Country-wide assessment of tick-borne pathogens collected in ticks between 2021 and 2024 in Germany, with a focus on *Francisella*: A one health pilot study

**DOI:** 10.1016/j.onehlt.2025.101190

**Published:** 2025-09-05

**Authors:** Kristin Köppen, Natalia Marta Zmarlak-Feher, Achim Dörre, Peter Hagedorn, Claudia Kohl, Klaus Heuner

**Affiliations:** a Working group: Cellular Interactions of Bacterial Pathogens, Centre for Biological Threats and Special Pathogens, Highly Pathogenic Microorganisms (ZBS 2), Robert Koch Institute, Berlin, Germany; b Mycotic and Parasitic Agents and Mycobacteria, Robert Koch Institute, Berlin, Germany; c ECDC Fellowship Programme, Public Health Microbiology Path (EUPHEM), European Centre for Disease Prevention and Control (ECDC), Stockholm, Sweden; d Department of Infectious Disease Epidemiology, Robert Koch Institute, Berlin, Germany; e Postgraduate Training for Applied Epidemiology, Robert Koch Institute, Berlin, Germany; f Center for Biological Threats and Special Pathogens 1 (ZBS 1), Robert Koch Institute, Berlin, Germany

**Keywords:** Ticks, Tick-borne disease, Tick-borne pathogens, *Francisella tularensis* subsp. *holarctica*, *Francisella*-like endosymbiont, Germany

## Abstract

Ticks are important vectors for several pathogens, among which *Francisella tularensis* subsp. *holarctica* is the most relevant tularaemia-causing subspecies in Europe. The number of human tularaemia cases in Germany has increased in recent years, and ticks play an important role in disease transmission. The aim of this study was to perform a pilot study addressing the presence of tick-borne pathogens in ticks across Germany, with a special focus on *Francisella*.

A total of 339 *Dermacentor reticulatus* ticks and 353 *Ixodes ricinus* ticks were collected in Germany between 2021 and 2024. DNA was extracted and analysed individually by multiplex qPCR assays detecting *F. tularensis* subsp. *holarctica*, *Francisella*-like endosymbionts, *Rickettsia* spp., *Borrelia burgdorferi* sensu lato complex, *B. miyamotoi*, *Anaplasma phagocytophilum*, *Ehrlichia* spp., *Coxiella burnetii*, *Bartonella* spp., *Babesia* spp., and tick-borne encephalitis virus.

PCR testing revealed a varying frequency of these pathogens depending on the tick species. The most frequently identified bacteria were *Francisella-*like endosymbionts (18–97 %), *Rickettsia* spp. (32–74 %), and *B. burgdorferi* (0–16 %). The occurrence of *F. tularensis* subsp. *holarctica*, *B. miyamotoi*, *A. phagocytophilum*, *Babesia* spp., and tick-borne encephalitis virus was observed at a low frequency in ticks (less than 10 % in either tick species). *Coxiella burnetii*, *Ehrlichia* spp., and *Bartonella* spp. were not detected in the investigated ticks. More than 70 % of *D. reticulatus* ticks and 19 % of *I. ricinus* ticks were positive for at least two pathogens. There was a significant co-occurrence of *Francisella-*like endosymbionts and *Rickettsia* spp. in both tick species. This pilot study offers a framework for the surveillance of common, rare, and newly emerging tick-borne pathogens in Germany.

## 1 Introduction

Ticks are vectors of multiple pathogens, including *Francisella* spp., *Borrelia* spp., and tick-borne encephalitis virus (TBEV), which cause tick-borne diseases, such as tularaemia, in humans and animals [1,2]. In Europe, the tularaemia-causing agent is *Francisella tularensis* subsp. *holarctica* (*Fth*), which affects both humans and animals [[3], [4], [5]]. The *Francisella* genus consists of more than 20 species, including pathogenic species (e.g., *F. tularensis* subspecies *tularensis*, *holarctica*, and *mediasiatica*; and fish pathogens, such as *F. noatunensis* and *F. orientalis* [6,7]), environmental species (e.g., *F*. *endociliophora* [8]), *Francisella*-like endosymbionts (FLEs) [9], and opportunistic species (e.g., *F*. *opportunistica* [10], *F. novicida* [11,12], *F. philomiragia* [11], and *F. hispaniensis* [13]), which can also induce tularaemia-like symptoms in immunosuppressed individuals. Tularaemia, also known as rabbit fever, has a diverse clinical manifestation in humans, including flu-like symptoms, skin lesions, and severely swollen lymph nodes [3,5]. Severe cases may lead to pneumonia and systemic infection, culminating in multi-organ failure, which may be fatal. In Germany, the number of reported human tularaemia cases has steadily increased in recent years, from 17 cases in 2011 to 214 cases in 2024 [14]. However, due to the similarity of tularaemia symptoms to clinical signs of other infections, tularaemia is often overlooked and, therefore, underdiagnosed [15]. Pathogen transmission to humans can occur through the consumption of contaminated food and water, contact with infected animals, or arthropod bites [[3], [4], [5]]. Ticks are important vectors for *F. tularensis*, and it has been estimated that 10–20 % of human cases in Europe, and thus in Germany, are transmitted by ticks [[16], [17], [18]]. In Europe, *Fth* has been identified in multiple tick species, including *Ixodes ricinus*, *Dermacentor reticulatus*, and *Haemaphysalis concinna* [9]. Based on previous studies, the prevalence of *Fth* in ticks is rather low, ranging from 0 to 8.4 % in Europe [9,[19], [20], [21], [22], [23], [24], [25]]. Thus far, the presence of *Fth* in ticks has only been investigated in specific areas of Germany [[20], [21], [22],^24^]. The prevalence of *Fth* throughout Germany remains unknown. In addition to *Fth*, FLEs are frequently found in ticks, such as *Dermacentor* spp., *Hyalomma* spp., and *Rhipicephalus sanguineus* [9,23,[25], [26], [27], [28]]. FLEs are genetically related to *F. tularensis*, having a high 16S rDNA sequence similarity. Therefore, using only 16S rDNA sequences to detect *F. tularensis* in ticks may result in false positives, as FLEs may be misidentified as *F. tularensis* [9,[28], [29], [30]].

In addition to *F. tularensis*, other tick-borne pathogens that cause different diseases, including Lyme disease, tick-borne encephalitis, anaplasmosis, and babesiosis, are present in Germany. Lyme disease (borreliosis) is the most widely spread tick-borne disease in Germany with 6000–14,000 human cases notified annually [1,2,14]. The clinical manifestations of borreliosis are diverse and can vary in severity [1,2]. However, flu-like symptoms and a bullseye rash (*Erythema migrans*) are commonly reported. In addition, the nervous system, joints, and heart can also be affected. The causative agent of Lyme disease, the *Borrelia burgdorferi* sensu lato complex, includes more than 20 genotypes, 6 of which are human pathogens and 5 of which have been described in Germany (*B. burgdorferi* senso stricto, *B. garinii*, *B. afzelii*, *B. spielmanii*, and *B*. *bavariensis*, [1,2,31]. Most of them are primarily associated with *I. ricinus*, in which the prevalence of *B. burgdorferi* sensu lato complex ranges from 5 to 50 % in Germany [1,2,24,[32], [33], [34], [35], [36], [37], [38], [39], [40], [41]].

*Borrelia miyamotoi* is an emerging tick-borne pathogen with the first reported human cases in Russia in 2011 [42]. Clinical signs caused by *B. miyamotoi* differ from those of *B. burgdorferi*, including relapsing fever [1,43]. Thus far, only one human case caused by *B. miyamotoi* has been documented in Germany [44], but *B. miyamotoi* has been identified in *I. ricinus* tick populations in Germany with a prevalence below 5 % [33,[35], [36], [37], [38],^41^,^43^,[45], [46], [47], [48]].

The second most common tick-borne disease in Germany is tick-borne encephalitis with approximately 300–700 cases annually [1,14]. In about 10 % of patients, the infection causes significant neurological symptoms. The causative agent, the TBEV, is primarily associated with *I. ricinus* but has also been found in other tick species, such as *D. reticulatus* [1]. The virus is typically found in defined, small, and stable foci, leading to a prevalence ranging from 0 to 3.6 % in Germany [1,[49], [50], [51], [52], [53]].

Another group of tick-borne pathogens include *Rickettsia* and *Anaplasma* within the order Rickettsiales [1,2]. The genus *Rickettsia* comprises more than 160 species and includes tick-, louse-, and flea-borne human pathogens and tick endosymbionts [54]. They are classified into four main groups: (1) spotted fever group, (2) typhus group, (3) transitional group, and (4) ancestral group. In Northern Europe and Germany, the predominant human pathogenic tick-borne species are *R. helvetica* and *R. monacensis*. Both belong to the spotted fever group and cause Mediterranean spotted fever-like symptoms, including fever and rashes, but organs, such as the heart and brain, can also be affected [1,54]. Here, *I. ricinus* serves as the main vector with a *Rickettsia* prevalence between 1 and 66 % [38,55,56]. *Rickettsia raoultii* and *R. slovaca* are also found in Germany but are primarily associated with *D. reticulatus*. Both species have been described as human pathogens [2].

Within the *Anaplasma* genus, *A. phagocytophilum* has the highest zoonotic potential causing tick-borne fever in ruminants and granulocytic anaplasmosis in humans [1,2]. This pathogen is primarily found in *I. ricinus*, and its presence shows seasonal and annual variations up to a presence of 30 % in tested ticks [24,[32], [33], [34],^36^,^57^]. Additionally, *A. phagocytophilum* has been identified in other tick species, including *D. reticulatus* (0.00–0.42 % in Germany [34,57,58]).

*Babesia* are tick-transmitted intraerythrocytic protozoan parasites that infect mammals and birds and have a high impact on farm and pet animal health worldwide [1,59]. More than 100 *Babesia* spp. have been identified, but only a few, including *B. divergens*, *B. microti*, and *B*. *venatorum*, are pathogenic to humans [59]. *Ixodes ricinus* is the main vector for these three emerging tick-borne *Babesia* spp., but *D. reticulatus* can also be infected [60]. The estimated presence of *Babesia* in ticks worldwide ranges from 1 to 4 % (in Germany, about 2 %).

Fluctuations in tick populations, driven by factors such as climate change, regarding both temperature and humidity, significantly affect tick distribution patterns, leading to geographical and seasonal shifts of tick presence throughout the year [[61], [62], [63]]. These changes affect tick populations and contribute to the expansion and emergence of tick-borne pathogens [64,65]. As ticks continue to expand into new regions and their activity periods shift, there is a growing need for comprehensive studies to better understand the pathogens they carry. The aim of this study was to implement a methodology to assess the presence of various tick-borne pathogens, with a particular focus on *Fth* in tick vectors. A new quantitative polymerase chain reaction (qPCR) multiplex assay has been used to distinguish between *Fth* and FLEs, which are genetically related and commonly found in ticks. This study provides a unique contribution by offering an updated, nationwide assessment of tick-borne pathogens in *I. ricinus* and *D. reticulatus*, two widely distributed tick species in Germany [66,67]. The study design provides a balanced 50:50 ratio of tick species in each federal state, enabling an analysis of the pathogens' presence within these species and their vector specificity. By testing multiple pathogens in individual ticks, this study also reveals potential co-occurrence patterns between pathogens.

## 2 Material & methods

### 2.1 Tick collection

Ticks were collected by volunteers across Germany between 2021 and 2024 as part of the citizen science project “Zecken und ihre Pathogene im Klimawandel” (ZEPAK, https://www.zepak-rki.de/; funded by the German Federal Ministry of Health). The voluntary collectors sent the ticks to the Robert Koch Institute (Berlin, Germany), where they were morphologically identified to the species level and stored individually at −80 °C until nucleic acid extraction. The study mainly analysed adult ticks (except for two larvae and six nymphs) that were both host-seeking (questing) and host-feeding. A total of 17–29 *I. ricinus* and 20–28 *D. reticulatus* ticks were analysed for each tested federal state (leading to an approximate ratio of 50:50), resulting 692 ticks (Supplementary Table 1). The federal state of Bremen was excluded from the study as neither *D. reticulatus* nor *I. ricinus* ticks were collected. In Hamburg, only *I. ricinus* ticks were collected.

### 2.2 Genetic material extraction

Ticks were analysed individually, and nucleic acid from individual ticks was extracted separately using the blackPREP Tick DNA/RNA Kit (IST Innuscreen GmbH, Berlin, Germany) and a SpeedMill homogeniser (Analytik Jena, Jena, Germany) according to manufacturer's instructions. The samples were eluted in 100 μL of double-distilled water and stored at −20 °C until further use.

### 2.3 Pathogen detection by quantitative real-time PCR

*Detection of Francisellaceae* spp., *Francisella tularensis* subsp. *holarctica*, *and Francisella-like endosymbiont.*

All primers and probes used in this study are listed in Supplementary Table 2. The F-16S target was used as recently described [68] to detect all members of the *Francisellaceae* family. *Francisella tularensis* subsp. *holarctica* was specifically targeted using the primer and probe (Fth-B2) described by Larson et al. [69]. Furthermore, a new primer/probe set was designed to specifically identify FLEs in ticks. For this reason, we selected the *tul* gene, which is commonly used for *F. tularensis* detection in qPCR assays [70]. A *tul* DNA sequence specific for FLE (and *F. persica*, which was isolated from a tick (*Argus* (*Persicargas*) *arboreus*) in Egypt, [71]) was chosen, and primer sequences (FLE-tul-F and FLE-tul-R) and probe (FLE-tul-P) were designed. They were initially tested in a duplex PCR reaction with the F-16S set (which detects all *Francisellaceae* strains, [68]). Duplex PCR was tested using tick DNA obtained from *D. reticulatus* and *I. ricinus* and a set of Francisellaceae strains of different genera, such as *F. tularensis* subsp. *holarctica*, *F. novicida*, *F*. *philomeragia*, *F. hispaniensis*, and *F. persica*. The FLE set was negative for all tested *Francisellaceae* strains, except for *F. persica*, as expected. Moreover, the FLE set showed an amplification curve for DNA samples obtained from *D. reticulatus* but was negative for *I. ricinus* samples. In selected samples, the presence of FLEs in ticks was confirmed by 16S rDNA amplification and subsequent DNA sequencing. Using the F-16S set, we verified that only the *D. reticulatus* samples were positive for *Francisellaceae*. To screen tick DNA samples, we implemented a multiplex panel consisting of F-16S, FLE-tul, and Fth-B2 sets to allow the detection of all strains of the *Francisellaceae* family, FLE, and *F. tularensis* subsp. *holarctica* (Supplementary Table 3). This panel was tested as described in Köppen et al. [68] with a large set of *Francisellaceae*, including all genera, such as *Allofrancisella*, *Francisella*, and *Pseudofrancisella*, and was found to be specific for the detection of *F. tularensis* subsp. *holarctica* and FLEs.

qPCR (Applied Biosystems TaqMan technology) was run using a total volume of 25 μL comprising 5 μL DNA, 6.25 μL TaqMan Environmental MasterMix 2.0 (ThermoFisher, Darmstadt, Germany), 10 μmol/μL primers (0.75 μL each), and 10 μmol/μL probes (0.25 μL each). Primers were obtained from Eurofins Genomics (Ebersberg, Germany) and probes from Metabion (Planegg/Steinkirchen, Germany). Samples were analysed in technical duplicates in each run. In one duplicate, the internal control (= KoMa plasmid [72], provided by the Centre for Biological Threats and Special Pathogens, Highly Pathogenic Microorganisms (ZBS 2)) was added (10^3^ genome equivalents per reaction). Amplification was performed utilising a Bio-Rad CFX96 cycler (Bio-Rad Laboratories, Feldkirchen, Germany) with the following cycle: initial denaturation at 95 °C for 10 min, followed by 40 cycles of denaturation at 95 °C for 15 s and a combined primer annealing and elongation step at 60 °C for 60 s.

*Detection of Rickettsia* spp., *Borrelia burgdorferi* sensu lato *complex*, *Borrelia miyamotoi*, *Anaplasma phagocytophilum*, *Ehrlichia* spp., *Babesia* spp., *Coxiella burnetii*, *Bartonella* spp., *and Tick-borne encephalitis virus.*

The Novaplex Tick-borne Disease Expanded Assay (Seegene, Duesseldorf, Germany) was used to simultaneously detect *Rickettsia* spp., *Borrelia miyamotoi*, *Borrelia burgdorferi* sensu lato complex, *Anaplasma phagocytophilum*, *Ehrlichia* spp., TBEV, *Babesia* spp., *Coxiella burnetii*, and *Bartonella* spp. The kit's enzyme mix contains a reverse transcriptase enabling the detection of the RNA of TBEV. The assay requires an internal control DNA, which was added to the samples' master mix but not to the control reactions (positive and negative controls). PCR was carried out according to the manufacturer's instructions using a CFX96™ Real-Time C1000 Cycler (Bio-Rad Laboratories, Feldkirchen, Germany), the CFX Maestro software (version 3.1, Bio-Rad Laboratories, Feldkirchen, Germany), and SeegeneViewer for RUO software (version 3, Seegene, Duesseldorf, Germany).

### 2.4 Validation of results by PCR and Sanger sequencing

Conventional PCR was performed to confirm the tick species of selected samples, as described by Beati et al. [73] and Hoffman et al. [74], with specific primers for *I. ricinus* and *D. reticulatus*, as described by Michelet et al. [19], with some modifications. All primers used in this study are listed in Supplementary Table 2. Briefly, tick DNA samples were diluted 1:10 in double-distilled water and used as template DNA for the PCR reaction, which consisted of 2.5 u/rxn Taq DNA Polymerase (Qiagen, Hilden, Germany), 1× PCR buffer, 0.2 mM dNTP mix, 0.1–0.5 μM primer each, and 1× Q solution. The PCR assays were run in a total volume of 50 μL using the thermal cycler Biometra TRIO-Thermoblock (Analytik Jena, Jena, Germany). PCR specific to *I. ricinus* and *D. reticulatus* was performed under the following conditions: initial DNA denaturation at 94 °C for 3 min; 35 cycles of denaturation at 94 °C for 30 s, annealing at 58 °C for 30 s, and extension at 72 °C for 30 s; and final extension at 72 °C for 10 min. For tick species identification, amplification was run under the following conditions: initial DNA denaturation at 94 °C for 3 min; 5 cycles of denaturation at 94 °C for 30 s, annealing at 51 °C for 1 min, and elongation at 68 °C for 45 s [73,74]; 25 cycles at 94 °C for 30 s, 53 °C for 1 min, and 70 °C for 45 s; and final extension at 72 °C for 10 min. PCR products were either checked for their size using gel electrophoresis (*I. ricinus-* and *D. reticulatus-*specific PCR) or purified using the Wizard® SV Gel and PCR Clean-Up System (Promega, Walldorf, Germany) according to the manufacturer's instructions, sequenced using Sanger sequencing, and analysed using BLAST.

### 2.5 Data analysis and statistics

Data analysis and visualisation were conducted in R version 4.3.0 using ‘tidyverse’ [75] and ‘forestplot’ [76]. Differences in pathogen presence between *D. reticulatus* and *I. ricinus* ticks were tested using Pearson's chi-squared test, and co-occurrence patterns were either assessed by Pearson's chi-squared test, where appropriate, or by Fisher's exact test.

## 3 Results

### 3.1 Tick collection

A total of 692 ticks collected by volunteers across Germany between 2021 and 2024 were used in this study. For each tested federal state, we selected 50 ticks consisting of an approximately equal number of *D. reticulatus* and *I. ricinus* specimens. The number of ticks collected from each federal state is shown in Supplementary Table 1. In total, 339 *D. reticulatus* ticks were collected and analysed from 14 of the 16 German federal states (city-states Bremen and Hamburg were excluded), and 353 *I. ricinus* ticks were collected from 15 of the 16 states (Bremen was excluded).

Analysis of collection dates revealed a seasonal variation in the presence of both tick species. *Ixodes ricinus* was predominantly collected in April and May (56.4 %), whereas *D. reticulatus* was mostly collected in late winter / early spring (February–March, 29.8 %) and late summer/early autumn (August–October, 41.0 %) (Supplementary Fig. 1). In terms of host origin, most ticks were collected from animals (59.3 % for *D. reticulatus* and 45.3 % for *I. ricinus*, Supplementary Table 4). A lower percentage of ticks was collected from humans (6.8 % for *D. reticulatus* and 23.2 % for *I. ricinus*), and a significant portion had an unknown host (33.9 % for *D. reticulatus* and 31.4 % for *I. ricinus*).

Regarding the animal hosts, *D. reticulatus* was mostly collected from dogs (71.6 %), whereas *I. ricinus* was nearly equally distributed between cats (48.8 %) and dogs (41.9 %, Supplementary Table 5). Other animal hosts comprised horses, boars, deer, goats, and rabbits. Regarding their sex distribution, both species were predominantly female (54.0 % for *D. reticulatus* and 70.0 % for *I. ricinus*, Supplementary Table 6), with 42.8 % of *D. reticulatus* and 27.2 % of *I. ricinus* being male. The sex remained undetermined in 3.2 % of *D. reticulatus* and 2.8 % of *I. ricinus*.

### 3.2 Identification of tick-borne pathogens within study population

**Fig. 1:**
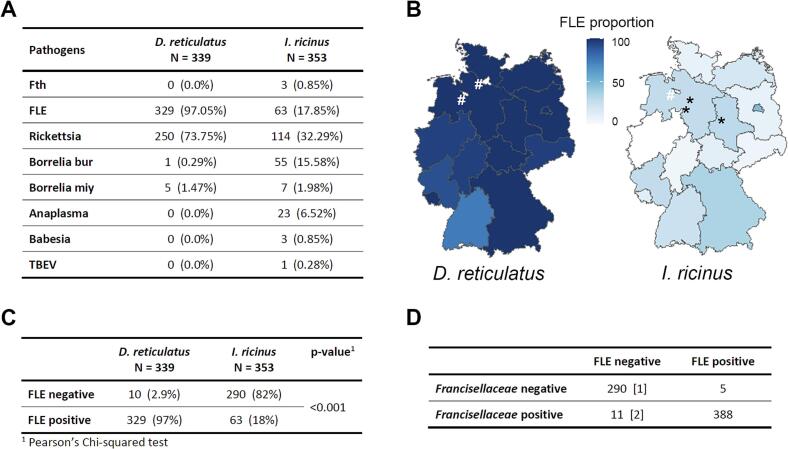
**Pathogen presence in ticks.***Francisella*-like endosymbiont (FLE) is predominantly found in *D. reticulatus* ticks, while *F. tularensis* subsp. *holarctica* (*Fth*) is rarely detected in ticks from Germany. **A.** Overview of pathogens detected in ticks across Germany. The number of tick individuals tested positive for each listed pathogen, by a tick species. The proportion of infected ticks over whole *D. reticulatus* or *I. ricinus* populations are indicated in the brackets. Abbreviations: Fth = *Francisella tularensis* subsp. *holarctica*, FLE = *Francisella*-like endosymbiont, Borrelia bur = *Borrelia burgdorferi* sensu lato complex, Borrelia miy = *Borrelia miyamotoi*, Rickettsia = *Rickettsia* spp., Anaplasma = *Anaplasma phagocytophilum*, Babesia = *Babesia* spp., TBEV = tick-borne encephalitis virus. **B.** Map of positive FLE tick proportions by federal state and tick species. Each asterisk represents an individual tick tested positive for *Fth*, indicating its federal state collection. Hash indicates the federal states where ticks of a particular species were not collected for screening. **C.** Comparison of FLE presence in tick species. FLE were detected more frequently in *D. reticulatus* than in *I. ricinus*. A Pearson's chi-squared test was performed to compare the numbers of positive and negative ticks within both species. **D.** Correlation between *Francisellaceae* and FLE testing results. The majority of ticks positive for *Francisellaceae* were also tested positive for FLE. The numbers in square brackets depict *Fth* positively tested ticks.

Pathogen testing revealed a varying frequency of pathogens depending on the tick species. Rare pathogens found in less than 10 % of either tick species included *Fth*, *B. miyamotoi*, *A. phagocytophilum*, *Babesia* spp., and TBEV (Fig. 1). *Coxiella burnetii*, *Ehrlichia* spp., and *Bartonella* spp. remained undetected in the collected ticks. FLE, *Rickettsia* spp., and *B. burgdorferi* s.l. were the dominant pathogens with more than 10 % of either tick species testing positive.

#### 3.2.1 *Francisella tularensis* subsp. *holarctica* and *Francisella*-like endosymbionts

*Fth* was detected in three *I. ricinus* ticks from Lower Saxony and Saxony-Anhalt (0.85 %, Fig. 1A, B). In contrast, FLE was found in all analysed federal states (Fig. 1B and Supplementary Fig. 2), with significantly more *D. reticulatus* ticks (97 %) testing positive than *I. ricinus* (18 %) (Fig. 1A, C). In *D. reticulatus*, FLE showed a percentage varying from 75 % in Baden-Wuerttemberg to 100 % in 9 of 14 federal states tested, and the proportion of *I. ricinus* ranged from undetectable (0 %) in North Rhine Westphalia to 48 % in Berlin (Supplementary Fig. 2). The assay detecting *Francisella* also identified 9 ticks that were negative for both FLE and *Fth* but positive for *Francisellaceae*, suggesting the presence of other *Francisellaceae* spp., which requires further investigation (Fig. 1D).

#### 3.2.2 Occurrence of other tick-borne pathogens

**Fig. 2:**
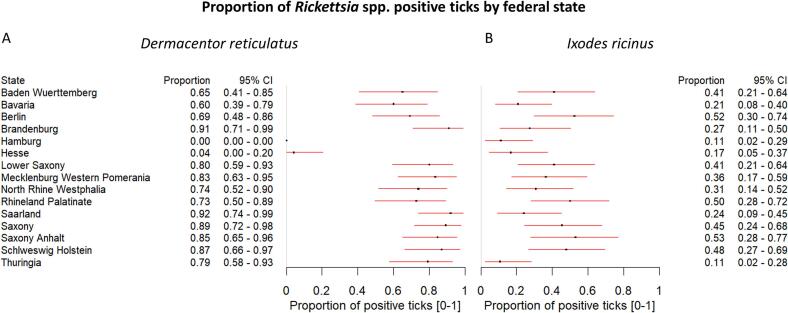
**Presence of *Rickettsia*** spp. **in *D. reticulatus* and *I. ricinus* ticks by federal state. A.** The forest plot shows the presence of *Rickettsia* spp. in *D. reticulatus* in all tested federal states, with a lower proportion of positive ticks observed in Hesse. The proportion of positively tested ticks is represented by a dot, with the 95 % confidence intervals (95 % CI) indicated by the red horizontal lines. Hamburg – no *D. reticulatus* tested. **B.** The forest plot illustrates that *Rickettsia* spp. is present in *I. ricinus* at similar levels across all federal states. The proportion of positively tested ticks is represented by a dot, with the 95 % confidence intervals (95 % CI) indicated by the red horizontal lines. (For interpretation of the references to colour in this figure legend, the reader is referred to the web version of this article.)

*Rickettsia* spp. were present in 250 *D. reticulatus* ticks (73.75 %) from all 14 federal states, with a percentage ranging from 60 % in Bavaria to 92 % in Saarland (Figs. 1A and 2A). There was a low positive tick presence in Hesse, where only 4 % of ticks were positive for *Rickettsia* spp. Further analysis revealed that most ticks from Hesse were collected from dogs (21/25, 84 %), but this did not explain the low prevalence of *Rickettsia* spp. in *D. reticulatus* ticks, even after stratification by dog as a host type and comparison with other federal states, as dogs were found to be the most prevalent host type (Supplementary Table 7). The overall proportion of *Rickettsia* spp.-positive *D. reticulatus* ticks was higher than for *I. ricinus*. For *I. ricinus*, the percentage of ticks that tested positive for *Rickettsia* spp. was 32.29 % (114 ticks) and ranged from 11 % in Hamburg and Thuringia to 53 % in Saxony Anhalt across the federal states with consistent presence in all 15 federal states (Fig. 2B). No regional variations were observed for *Rickettsia* spp. in *I. ricinus*.

**Fig. 3:**
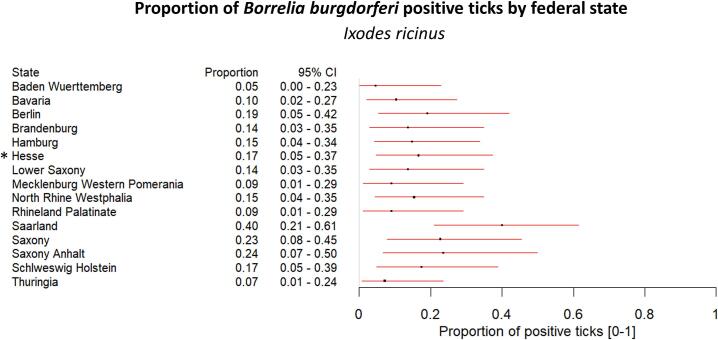
**Presence of *Borrelia burgdorferi* s.** l. **in *I. ricinus* ticks by federal state.** The forest plot illustrates that *Borrelia burgdorferi* s. l. is present in all federal states at similar levels in *I. ricinus* ticks. The proportion of positively tested ticks is represented by a dot, with 95 % confidence intervals (95 % CI) indicated by the red horizontal lines. The black asterisk (*) marks the location of a *D. reticulatus* tick tested positive for *Borrelia burgdorferi* s. l.. (For interpretation of the references to colour in this figure legend, the reader is referred to the web version of this article.)

*Borrelia burgdorferi* s.l. was predominantly found in *I. ricinus* ticks (15.58 %), with only one *D. reticulatus* tick testing positive (0.29 %, Fig. 1A). The tick species was confirmed by PCR and sequencing (see Material & Methods). The pathogen was present in less than half of the tested *I. ricinus* ticks across all federal states (prevalence ranging from 5 % in Baden-Wuerttemberg to 40 % in Saarland, Fig. 3). No regional variation was observed for *B. burgdorferi* in *I. ricinus*. Additionally, no significant correlation was observed between the percentage of *B. burgdorferi*-positive ticks and human Lyme disease surveillance data for Germany between 2021 and 2024 (Supplementary Fig. 3).

**Fig. 4:**
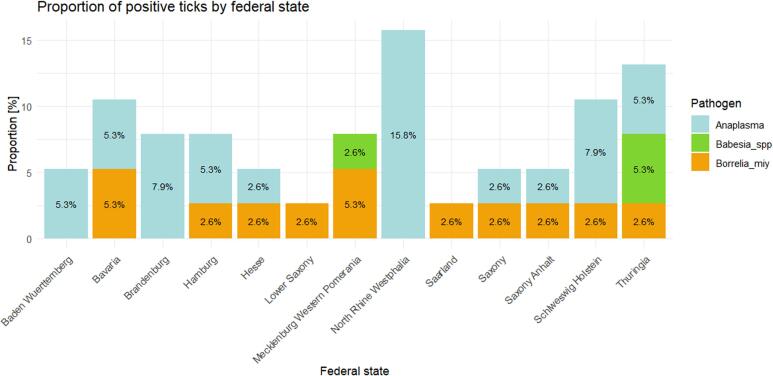
**Presence of *Anaplasma phagocytophilum*, *Babesia*** spp. **and *Borrelia miyamotoi* in ticks.** The histogram displays the proportion (in %) of ticks tested positive for *Anaplasma phagocytophilum* (Anaplasma), *Babesia* spp. (Babesia_spp) and *Borrelia miyamotoi* (Borrelia_miy), across the entire tick population (*D. reticulatus* and *I. ricinus*) for each federal state.

Other pathogens detected in our study included the emerging pathogen *B. miyamotoi*, which was found in low proportion in seven *I. ricinus* (1.98 %) and five *D. reticulatus* ticks (1.47 %) but was consistently present in most federal states (Fig. 1, Fig. 4). For *D. reticulatus*, the tick species was additionally confirmed by PCR and Sanger sequencing. *Anaplasma phagocytophilum* was detected in 23 *I. ricinus* ticks (6.25 %) and in 10 of the 15 federal states tested, with the highest prevalence observed in North Rhine Westphalia (12.5 %; Fig. 1, Fig. 4). *Babesia* spp. were found in *I. ricinus* ticks from Thuringia and Mecklenburg-Western Pomerania at very low frequencies (0.85 %, Fig. 1, Fig. 4). In contrast, neither *A. phagocytophilum* or *Babesia* spp. was found in *D. reticulatus* ticks. Lastly, TBEV was detected in a single *I. ricinus* tick (0.28 %) collected from Thuringia (Fig. 1A). Our study did not detect *Ehrlichia* spp., *Bartonella* spp., or *Coxiella burnetii* in the tested ticks.

### 3.3 Pathogen co-occurrence

Regarding pathogen co-occurrence patterns within tick vectors, 73.2 % of *D. reticulatus* ticks and 20.4 % of *I. ricinus* ticks tested positive for two or more pathogens (Supplementary Table 8). The number of pathogens detected per tick was comparable across sexes (Supplementary Table 9). In both tick species, co-occurrence analysis revealed a significant association between FLE and *Rickettsia* spp. (Table 1), but no other significant co-occurrence patterns were observed among other pathogens (data not shown).

**Table 1:** Co-occurrence patterns of *Francisella*-like endosymbionts (FLE) and *Rickettsia* spp. (Rcke) in *Dermacentor reticulatus* and *Ixodes ricinus* ticks.^a^, ^b^

	*D. reticulatus*		*I. ricinus*	
	**FLE negative** ***N* = 10**	**FLE positive** ***N* = 329**	**FLE negative** ***N* = 290**	**FLE positive** ***N* = 63**
Rcke negative	7 (70 %)	82 (25 %)	214 (74 %)	25 (40 %)
Rcke positive	3 (30 %)	247 (75 %)	76 (26 %)	38 (60 %)
*p*-value	0.004^a^		<0.001^b^	

Ticks surveyed for FLE and *Rickettsia* spp. were analysed for significant co-occurrence patterns using Fisher's exact test for *D. reticulatus* and Pearson's chi-squared test for *I. ricinus*.

a **Fisher's exact test.**

b **Pearson's chi-squared test.**

## 4 Discussion

Ticks are known vectors of pathogens that pose significant risks to both human and animal health. In this pilot study, we developed a methodology for pathogen surveillance in ticks. We focused on two tick species in Germany, *I. ricinus* and *D. reticulatus*, which are widely distributed across the country [66,67]. The ticks used in this research were collected through the citizen-science project ZEPAK (https://www.zepak-rki.de/), which has the advantage of encompassing a broad, randomly selected range of locations. This countrywide initiative facilitated tick collection across Germany, excluding Hamburg (where *D. reticulatus* was not available) and Bremen (where neither species was available). The scope of this nationwide pilot study provides insights into the presence of tick-borne pathogens across Germany, offering an advantage over previous studies, which were often limited to specific regions [21,24,32,34,35,37,38,40,41,46,[77], [78], [79], [80], [81]]. Moreover, working with society-driven projects builds an awareness not only among professionals but also within society. The weaknesses of such projects are that the researchers have limited influence on material collection (exact locations ticks are obtained might be caused by collector preferences) as well as material handling during the collection process, which might affect the stability of genetic material and, thus, downstream molecular analysis.

Our findings are consistent with previous studies reporting seasonal variation in tick activity [82]. As expected, the data revealed clear seasonal patterns in the collection months for both *I. ricinus* and *D. reticulatus*, highlighting the seasonal nature of tick occurrence. *Dermacentor reticulatus* was mainly collected in February and March (29.8 %) and August–October (41 %), whereas *I. ricinus* was mainly collected in April and May (56.4 %). Information about seasonality is essential for understanding tick population dynamics and, therefore, associated pathogen transmission, further emphasising the importance of considering temporal factors in surveillance and vector control efforts.

FLEs were the most dominant bacteria found in all federal states in which ticks were collected, with *D. reticulatus* (97.05 %) being significantly more positive tested than *I. ricinus* (17.85 %). Differing proportions between the two tick species have been observed in other European countries [23,82,83]. However, the FLE proportion in *I. ricinus* ticks was lower compared to the data presented here (0.0–10.4 % vs. 17.9 %). In contrast, a higher frequency of FLE has been observed in *D. reticulatus* ticks, albeit with considerable variations, resulting in a prevalence ranging from 15 to 100 % [21,23,82,84,85]. FLEs are endosymbionts of various tick species, and they are more closely related to *F. tularensis* than to other *Francisella* spp., such as *F. philomiragia* or *F. hispaniensis* [9,[86], [87], [88], [89]]. It is unclear whether FLEs infect humans and cause disease [9]. The closely related species *F. persica* was isolated from a tick (*Argus* (*Persicargas*) *arboreus*) in Egypt [71], originally described as *Wolbachia persica* and later renamed and reclassified as *Francisella* [90]. *Francisella persica* has been shown to be pathogenic to hamsters and guinea pigs [71,89,91]. Therefore, *F. persica* may represent a “bridge organism” between FLEs and opportunistic *Francisella* spp. [88,90,92]. This highlights the need for knowledge on the presence of FLE in ticks across Germany.

FLEs exhibit a high 16 s rDNA similarity to *F. tularensis*, and if only 16 s rDNA is used to detect *F. tularensis*, ticks may be misidentified as positive for *F. tularensis*, leading to falsely high frequencies, as shown by Kugeler et al. and others [[28], [29], [30],^93^]. Therefore, the accurate and specific discrimination of FLE and *F. tularensis* in ticks is highly important. In the present study, a qPCR assay was utilised to detect three specific targets: (1) the 16 S rDNA target was employed to identify all representatives of the *Francisellaceae* family; (2) the FLE-tul gene was used for the specific identification of FLEs; and (3) the FTS_0806 target was used for *Fth* identification, as demonstrated by Larson et al. [69]. In our study, the frequency of *Fth*-positive ticks was very low (0.85 %), and only three *I. ricinus* ticks tested positive. These findings are consistent with other studies investigating the prevalence of *Fth* in ticks in Germany (rates range between 0 and 8.4 % [[20], [21], [22],^24^,^94^]. Two *Fth-*positive ticks were collected in Lower Saxony and one in Saxony-Anhalt. In consideration of the limited number of tested ticks and positive samples, it is not yet possible to make a definitive statement regarding a potential correlation between *Fth*-positive tested ticks and human tularaemia cases. However, several human cases have been reported describing tick-associated tularaemia in Germany [[95], [96], [97]], and a recent publication stated that approximately 20 % of human tularaemia cases were associated with ticks in the last 10 years in Baden-Wuerttemberg (Germany, [18]). Saxony-Anhalt exhibited an average tularaemia incidence of 0.32 per 100,000 inhabitants over the past 3 years (approximately 7 tularaemia cases per year), which is comparable to that of other federal states with a high tularaemia incidence, such as Bavaria and Baden-Wuerttemberg, where the average incidence per year is about 0.3 with approximately 34 cases observed per year in the last 3 years [14]. However, Lower-Saxony exhibits a low incidence (0.03), that suggesting that even in a low-incidence federal state, positive *Fth* ticks can be found in nature. Furthermore, in localised areas where tick-borne cases of tularaemia in humans and wildlife are frequent, the proportion of *Fth* may be elevated in ticks compared to other regions [20]. These areas merit investigation in future studies.

In the study population, we identified nine ticks that were positive for the *Francisellaceae* marker (F-16S, see Material & Methods and [68]), but negative for *Fth* and FLE markers. These ticks might carry other species of the *Francisellaceae* family. A case report of human infection with *F. philomiragia*, which may have been transmitted by an arthropod, has been documented in France [98]. However, less is known about the presence of *Francisellaceae* in ticks in Germany, highlighting the need for further research in this field.

The second most common bacteria found in our tick collection was *Rickettsia* spp., which showed a higher proportion in *D. reticulatus* (73.75 %) than in *I. ricinus* (32.29 %). A tick species-dependent pattern has also been observed in other German studies, which have reported a prevalence of *Rickettsia* between 43 and 82 % in *D. reticulatus* [32,34,79,80] and between 2 and 61 % in *I. ricinus* [24,32,34,36,80,99,100]. *Rickettsia* spp. are a diverse group of endosymbionts and pathogens that cause mild to severe disease in humans [1,2]. In Germany, several *Rickettsia* spp., have been found in ticks including *R. helvetica* and *R. monacensis*, which are associated with *I. ricinus*, and *R. raoultii* and *R*. *solvaca*, which are mainly associated with *D. reticulatus* [2]. All of these *Rickettsia* spp. can cause disease in humans. *Rickettsia* spp. were not identified here. Therefore, we can only speculate about the potential pathogenic impact of the high *Rickettsia* proportion observed in *I. ricinus* and *D. reticulatus* ticks. Still, the pathogenic potential should not be underestimated, as other German studies almost exclusively identified human-pathogenic *R. helvetica* within their *Rickettsia*-positive *I. ricinus* tick samples [36]. *Ricksettia monacensis*, *R. raoultii*, and *R*. *solvaca* have also be found in Germany [38,80,101,102]. Since these pathogens, *R. raoultii* and *R*. *solvaca*, can be transmitted by *D. reticulatus*, tick-transmitted rickettsiosis may also occur in the colder season. Although our study was limited to approximately 50 ticks per tested federal state, resulting in wide confidence intervals, the distribution of *Rickettsia* spp.-positive ticks was relatively consistent across most federal states (ranging from 11 % in Thuringia and Hamburg to 53 % in Saxony Anhalt for *I. ricinus* and from 60 % in Bavaria to 92 % in Saarland for *D. reticulatus*). However, the federal state of Hesse exhibited a significantly lower proportion of *Rickettsia* spp. in *D. reticulatus* ticks (4 %). Further analysis revealed that most ticks in Hesse (84 %) were collected from dogs. However, this observation alone is insufficient to explain the significantly low proportion of *Rickettsia* spp. in *D. reticulatus* ticks since the comparison with other federal states, where most ticks were also collected from dogs, did not show significant differences. Thus far, no explanation has been found for this low proportion. Although the small sample size limits our ability to draw firm conclusions, the unusually low proportion observed in Hesse warrants further investigation.

*Borrelia burgdorferi* s.l. complex was strongly associated with *I. ricinus* in the tested ticks, with a proportion of 15.58 %. In contrast, only one *D. reticulatus* tick was positive for *B. burgdorferi* (0.29 %). These findings are consistent with those reported in other studies from Germany, although the percentage of *B. burgdorferi*-positive *I. ricinus* ticks varied between 5 and 50 % [24,[32], [33], [34], [35], [36], [37], [38], [39], [40]]. We also found *B. burgdorferi*-positive *I. ricinus* ticks in 10 of 15 German federal states, with the proportions ranging from 5 % in Baden-Wuertemberg to 40 % in Saarland. Hence, no regional variation was observed for *Borrelia burgdorferi* s.l. in the studied tick population. The proportion of positive *I. ricinus* does not correlate with the incidence of human borreliosis. In addition to the already discussed limited tick sample size, these discrepancies between human and tick data may be partially attributed to underdiagnosis in human cases, underscoring the importance of robust tick surveillance for a more accurate assessment of infection risk. Variations in diagnostic practices and reporting protocols across federal states introduce additional complexity. Additionally, the notification of a human case from a specific federal state does not necessarily reflect the location of infection, as patients may have acquired the infection elsewhere. Therefore, these factors can lead to data biases, which may elucidate the absence of a discernible correlation between our tick and human surveillance data.

*Borrelia miyamotoi* is an emerging tick-borne pathogen causing the rare *B. miyamotoi* disease in humans [1]. Thus far, it has been shown to be strongly associated with *I. ricinus* ticks in Germany, with prevalence at rather low levels below 5 % [33,[35], [36], [37], [38],^41^,^43^,[45], [46], [47], [48]]. In 2019, *B. miyamotoi* was first found in *D. reticulatus* in Berlin, Germany [79]. In our study, the tick population exhibited a low proportion of *B. miyamotoi*, but *I. ricinus* and *D. reticulatus* ticks were almost equally positive for *B. miyamotoi* (1.98 % and 1.47 %, respectively). The morphological tick species identification was confirmed by two molecular methods, namely specific PCR assays [19] and 12S sequencing [73,74]. The detection of *B. miyamotoi* in *D. reticulatus* ticks in 10 federal states shows that the pathogen may have expanded its natural reservoir and distribution pattern, which may be important, as disease symptoms are often non-specific and serological tests for borreliosis do not detect infection with *B. miyamotoi* [44]. Although clinical reports of human infection with *B. miyamotoi* are rare, the clinical manifestations can be severe, particularly in immunocompromised individuals [1,44,45]. Thus, prospective studies and increased awareness are required to understand the potential risk of this emerging pathogen.

*Anaplasma phagocytophilum* was detected in 6.25 % of *I. ricinus* ticks but not detected in *D. reticulatus*. The highest proportion was found in North Rhine-Westphalia (12.5 %), but the pathogen was detected in 10 of the 15 federal states tested. Therefore, it is almost distributed throughout Germany. *Ixodes ricinus* represents the main vector for *A. phagocytophilum*, even though other tick species, such as *D. reticulatus*, can also be infected [34,57,58]. However, the prevalence of *A. phagocytophilum* is subject to seasonal and annual fluctuations, leading to proportions of up to 30 % [24,[32], [33], [34],^36^,^57^]. In the context of the one health concept, an understanding of the distribution of *A. phagocytophilum* is significant since it affects humans and domestic animals, such as cats, dogs and horses [1,2].

The protozoan parasites *Babesia* spp. showed a low proportion in the present tick collection, only 0.58 % of *I. ricinus* ticks and none of the *D. reticulatus* ticks were positive. This is consistent with other studies that have also shown an association between *Babesia* and *I. ricinus*, although some studies have reported higher prevalence rates (up to 18 % [24,32,34,57,99,100]. In contrast, a world-wide meta-analysis demonstrated that the prevalence of *Babesia* spp. decreased in recent years from 4.3 % (1992–1997) to 0.9 % (2015–2020) [60]. More than 100 *Babesia* spp. have been described, some of which are pathogenic to humans, such as *B. divergens* (causative agent of bovine babesias in cattle), *B. microti* and *B*. *venatorum* [59]. *Babesia divergens* and *B*. *venatorum* are only found in a small proportion of *I. ricinus* ticks in Germany, whereas *B. microti* can reach prevalence rates up to 5 % [38,77]. Moreover, *B. canis*, the causative agent of dog babesiosis in Europe, is primarily transmitted by *Dermacentor* spp. and causes mild to severe disease depending on the age and immune status of the affected dog [59]. In Germany, *D. reticulatus* ticks possess a low frequency of *B. canis* [103]. In this study, the proportion of all named human and animal pathogenic *Babesia* spp. in Germany was relatively low. However, the public health concern posed by *Babesia* spp. should not be overlooked.

In the tick population, we identified a single *I. ricinus* tick collected in Thuringia that was carrying TBEV. Further investigations may be conducted for a more comprehensive evaluation of this finding, such as strain differentiation. TBEV is known to be only found in small, defined and stable foci, and therefore, its prevalence varies between 0 and 3.6 % in Germany [1,[49], [50], [51], [52], [53]]. The area where the tick was collected (postal code 99092, Marbach (Erfurt)) has not yet been considered a TBEV-risk area [104], but it is located next to one. This finding serves as an indicator, and further studies in this field are needed.

Only in 2.1 % of *D. reticulatus* ticks was no pathogen detected, and most ticks (71.7 %) were positive for at least two pathogens. In comparison, 45.6 % of *I. ricinus* ticks were negative for all pathogens, and 19.3 % were positive for at least two pathogens. In both *D*. *reticultus* and *I. ricinus*, a significant co-occurrence of *Rickettsia* spp. and FLE was detected. Zając et al. also observed co-infection with *Rickettsia* spp. and FLE in *D. reticulatus* but not in *I. ricinus* [82]. It would be interesting to determine which *Rickettsia* spp. are co-infected with FLEs and whether they are the same for *I. ricinus* and *D. reticulatus*. These questions should be addressed in future studies. In general, ticks have the capacity to carry multiple pathogens, which can potentially result in co-infections in humans.

### 4.1 Conclusion & perspectives

This pilot study provides an overview of the presence of tick-borne pathogens across Germany, utilising a citizen science approach to facilitate broad, random sampling across the country. Our findings serve as a valuable pilot for pathogen surveillance in ticks, revealing the presence of both common and rare pathogens, such as *Rickettsia* spp., *B. burgdorferi* s.l., and *Fth*, and emerging threats, including *B. miyamotoi*. The methodology presented in this study offers a robust framework for future surveillance aiming to monitor tick-borne diseases, particularly in understanding the geographic distribution and pathogen prevalence in different regions. Future studies could build upon this work by including an increased tick sample size and separate investigations of pathogens found in ticks collected from animals and questing ticks. Additionally, future studies could narrow the focus to specific geographic locations, which would allow for more detailed risk assessments and a better understanding of local pathogen hotspots. Researchers can enhance the precision of risk area identification by mapping the exact locations of tick samples and correlating this data with environmental factors, such as climate, vegetation, and land use. This would be particularly beneficial in tracking the spread of rare pathogens, such as *Fth* and TBEV, which may be more localised and harder to detect at a broader level. In addition, future research could integrate tick density data with pathogen surveillance to model the burden of tick-borne diseases in areas where pathogens are prevalent. Understanding the relationship between tick density and the presence of specific pathogens would provide valuable insights into the dynamics of disease transmission risk assessment.

As environmental changes continue to influence tick populations and pathogen dynamics, future studies should focus on the long-term monitoring of tick distribution patterns in relation to climate change. Researchers can better predict and mitigate public health risks associated with tick-borne diseases by identifying shifting trends in tick populations and pathogen spread. This study provides a foundation for ongoing surveillance efforts that will be essential for managing the evolving landscape of tick-borne pathogens.

## CRediT authorship contribution statement

**Kristin Köppen:** Writing – original draft, Validation, Investigation, Formal analysis, Data curation. **Natalia Marta Zmarlak-Feher:** Writing – original draft, Validation, Investigation, Formal analysis, Data curation. **Achim Dörre:** Writing – review & editing, Methodology, Formal analysis, Data curation. **Peter Hagedorn:** Writing – review & editing, Methodology, Data curation. **Claudia Kohl:** Writing – review & editing, Supervision. **Klaus Heuner:** Writing – review & editing, Supervision, Funding acquisition, Conceptualization.

## Funding

This work received financial support from Robert Koch Institute and by the 10.13039/501100003107Federal Ministry of Health based on a resolution of the German Bundestag (2522PAT006). The ZEPAK project is funded by the Federal Ministry of Health based on a resolution of the German Bundestag (2520FSB402, BAB D81915). The author is a fellow of the ECDC Fellowship Programme, supported financially by the 10.13039/501100000805European Centre for Disease Prevention and Control (ECDC). The views and opinions expressed herein do not state or reflect those of the ECDC. The ECDC is not responsible for the data and information collation and analysis and cannot be held liable for conclusions or opinions drawn.

## Declaration of competing interest

The authors declare no conflicts of interest.

## Data Availability

Data will be made available on request.

## Appendix A Supplementary data

Supplementary material Image 1
