# Supplementary material for: Country-wide assessment of tick-borne pathogens collected in ticks between 2021 and 2024 in Germany, with a focus on Francisella: A one health pilot study
Source: One Health. 2025 Sep 5;21:101190. doi: 10.1016/j.onehlt.2025.101190 (PMC12452886; doi:10.1016/j.onehlt.2025.101190)
Supplement: Supplementary file 1 — Supplementary material [file mmc1.pdf]

Supplementary Material

Supplementary Figures

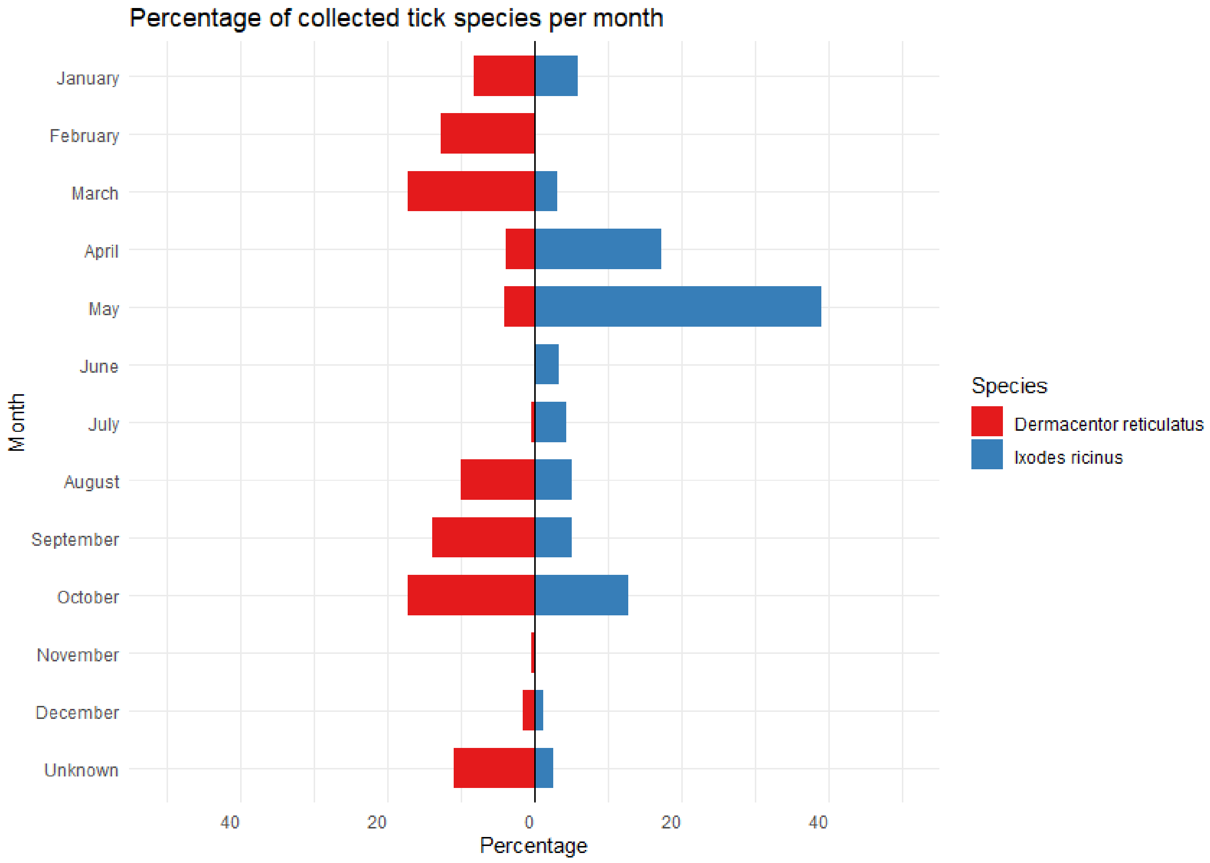

Supplementary Figure 1: Percentage of collected tick species per month. The proportion of tick individuals included in the analysis is listed by month of collection per *D. reticulatus* and *I. ricinus* species.

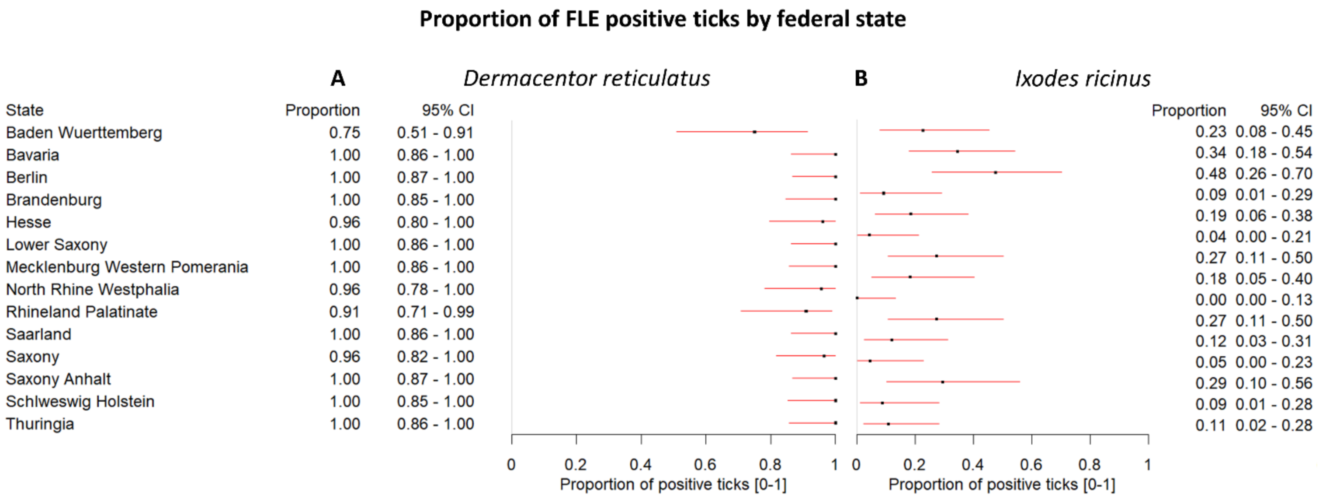

Supplementary Figure 2: Presence of FLE in *D. reticulatus* and *I. ricinus* ticks by federal state. The forest plot shows the presence of FLE in *D. reticulatus* (**A**) and *I. ricinus* (**B**) in all tested federal states. The proportion of positively tested ticks is represented by a dot, with the 95% confidence intervals (95% CI) indicated by the red horizontal lines. Hamburg – no *D. reticulatus* tested.

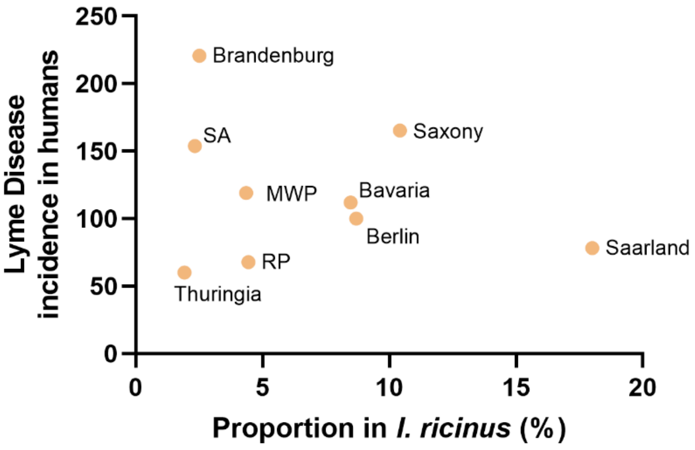

Supplementary Figure 3: Comparison between proportion of *B. burgdorferi* in *I. ricinus* ticks per federal state and Lyme disease surveillance data from human (notified between 2021 and 2024 [Survstat@RKI 2.0]). MWP, Mecklenburg-Western Pomerania; SA, Saxony-Anhalt; RP, Rhineland-Palatinate.

Supplementary Tables

Supplementary Table 1: Number of ticks used in the study by federal state, by tick species. The number of tick individuals included in the analysis is listed for each federal state. The percentage in brackets indicates the contribution of each tested species within each federal state.

Federal State	*D. reticulatus*	*I. ricinus*
**Baden Wuerttemberg**	20 (47.6%)	22 (52.4%)
**Bavaria**	25 (46.3%)	29 (53.7%)
**Berlin**	26 (55.3%)	21 (44.7%)
**Brandenburg**	22 (50.0%)	22 (50.0%)
**Hamburg**	0 (0.0%)	27 (100.0%)
**Hesse**	25 (51.0%)	24 (49.0%)
**Lower Saxony**	25 (53.2%)	22 (46.8%)
**Mecklenburg Western Pomerania**	24 (52.2%)	22 (47.8%)
**NA**	1 (50.0%)	1 (50.0%)
**North Rhine Westphalia**	23 (46.9%)	26 (53.1%)
**Rhineland Palatinate**	22 (50.0%)	22 (50.0%)
**Saarland**	25 (50.0%)	25 (50.0%)
**Saxony**	28 (56.0%)	22 (44.0%)
**Saxony Anhalt**	26 (60.5%)	17 (39.5%)
**Schleswig Holstein**	23 (50.0%)	23 (50.0%)
**Thuringia**	24 (46.2%)	28 (53.8%)
**Total**	339 (49.0%)	353 (51.0%)

Supplementary Table 2: Oligonucleotides and probes sequences for real time PCR assay.

PCR assay	Name	sequence (5´-> 3´)	target gene	description	reference
**Fth-B2**	Fth-B2-F	cctatccaatactccgagttagt	FTS_08 06	*Fth*-specific	Larson et al., 2020
	Fth-B2-R	aaatcaaaagaagagttaaaacaagc			
	Fth-B2-P	FAM-ctctggccagttatttttatcaaagccag-BHQ-1			
**F-16S**	F-16S-F2	tgacaggtgctgcacggctgt	16S rDNA	*Francisellaceae*- specific	Köppen et al., 2024
	F-16S-R2	gcagccctctgtaatacccatt			
	F-16S-P2	Cy5.5-acccaacttaatgatggtaactatcaatag-BHQ-3			
**FLE-tul**	FLE-tul-F	acaatggcaagctcctgaagg	tul4	FLE-specific	this study
	FLE-tul-R	ctacattagctgtccatttacca			
	FLE-tul-P	Cy5-ttgttgccttgcttaactgtcacagtt-BHQ-2			
**KoMa**	KoMa2-F	ggtgatgccgcattattactagg		internal amplification control	Kirchner et al., 2010
	KoMa2-R	ggtattagcagtcgcaggctt			
	KoMa2-P	JOE-ttcttgcttgaggatctgtcgtggatcg-BHQ-1			
**tick**	T1B	aaactaggattagatacc ct	12S rDNA	tick species identification	Beati et al., 2001; Hoffman et al., 2022
	T2A	aatgagagcgacgggcgatgt			
**I-ricinus**	Ix_ri_ITS2_F	cgaaactcgatggagacctg	ITS2	*I. ricinus* specific	Michelet et al., 2014
	Ix_ri_ITS2_R	atctccaacgcaccgacgt			
**D-reticulatus**	De_re_ITS2_F	aacccttttccgctccgtg	ITS2	*D. reticulatus* specific	Michelet et al., 2014
	De_re_ITS2_R	ttttgctagagctcgacgtac			

Supplementary Table 3: Discrimination of *F. tularensis* ssp. *holarctica*, FLE and other members of the *Francisellaceae* family by qPCR.

Sample	Target<br>F-16S	 FLE-tul	 Fth-B2
***Fth* positive**	+	-	+
**FLE positive**	+	+	-
**Other *Francisellaceae***	+	-	-
**No *Francisellaceae***	-	-	-

+: Ct value < 40; -: no Ct value

Supplementary Table 4: Characterization of tick hosts by tick species. The origin of each tick collected is categorized as animal, human, or NA, with data presented for each tick species.

	Animal	Human	NA
D. reticulatus	201 (59.3%)	23 (6.8%)	115 (33.9%)
I. ricinus	160 (45.3%)	82 (23.2%)	111 (31.4%)

Supplementary Table 5: Characterization of animal host species by tick species. For hosts categorized as animals in Supplementary Table 3, the specific species are further detailed. The percentage indicates the contribution of each animal host species within each tick species.

	Boar	Cat	Deer	Dog	Goat	Horse	Rabbit
D. reticulatus	17<br>(8.5%)	2<br>(1.0%)	0<br>(0.0%)	144<br>(71.6%)	11<br>(5.5%)	27<br>(13.4%)	0<br>(0.0%)
I. ricinus	0<br>(0.0%)	78<br>(48.8%)	10<br>(6.2%)	67<br>(41.9%)	3<br>(1.9%)	1<br>(0.6%)	1<br>(0.6%)

Supplementary Table 6: Characterization of tick sex by tick species.

	Female	Male	NA
D. reticulatus	183 (54.0%)	145 (42.8%)	11 (3.2%)
I. ricinus	247 (70.0%)	96 (27.2%)	10 (2.8%)

Supplementary Table 7: Analysis of *Ricketsia* spp. (Rcke) presence in *D. reticulatus* in Hesse and Saxony Anhalt or Thuringia and stratified by dog host.

	Global		Stratified by dog	
**Federal State**	**Rcke positive**	**Rcke negative**	**Rcke positive**	**Rcke negative**
**Hesse**	24 (96%)	1 (4%)	20 (95.24%)	1 (4.76%)
**Saxony Anhalt**	4 (15.38%)	22 (84.62%)	4 (18.18%)	18 (81.82%)
**Thuringia**	5 (20.83%)	19 (79.17%)	3 (23.08%)	10 (76.92%)

Supplementary Table 8: Number of pathogens per tick, by a tick species. The table presenting the number of pathogens tested positive, stratified by tick species, indicating the count of ticks tested positive for 0, 1, 2, 3, or 4 pathogens simultaneously. The percentages represent the proportion of ticks tested positive for each category relative to the total number of ticks within each species.

Nr of pathogens	*D. reticulatus*	*I. ricinus*
**0**	7 (2.1%)	161 (45.6%)
**1**	84 (24.8%)	120 (34.0%)
**2**	243 (71.7%)	68 (19.3%)
**3**	5 (1.5%)	3 (0.8%)
**4**	0 (0.0%)	1 (0.3%)

Supplementary Table 9: Number of pathogens per tick, by a tick sex.

Nr of pathogens	Female	Male	NA
**0**	118 (27.4%)	45 (18.7%)	5 (23.8%)
**1**	122 (28.4%)	75 (31.1%)	7 (33.3%)
**2**	186 (43.3%)	118 (49.0%)	7 (33.3%)
**3**	3 (0.7%)	3 (1.2%)	2 (9.5%)
**4**	1 (0.2%)	0 (0.0%)	0 (0.0%)
